# Black Ginseng–Deer Antler Peptide Composite Emulsion: Antimelanogenic and Antioxidant Effects In Vitro

**DOI:** 10.3390/foods15203665

**Published:** 2026-10-15

**Authors:** Hanmin Xie, Tingting Hu, Wanting Zhang, Huizhen Fang, Xin Qi, Chengbi Cui

**Affiliations:** 1 Agricultural College, Yanbian University, Yanji 133000, China; 2 School of Pharmacy, Yanbian University, Yanji 133000, China

**Keywords:** black ginseng, deer antler peptides, emulsion, antioxidant activity, antimelanogenic activity

## Abstract

This study aimed to develop a black ginseng–deer antler peptide composite emulsion (BGD) with antimelanogenic and antioxidant activities and to investigate its potential biological effects. The BGD formulation was optimized through response surface methodology, followed by assessment of its physicochemical characteristics, stability, preliminary skin safety, and in vitro antioxidant capacity. Its antimelanogenic and antioxidant effects were further investigated using α-melanocyte-stimulating hormone (α-MSH)-induced melanogenesis and H_2_O_2_-induced oxidative stress models in B16F10 cells, respectively. BGD showed good stability, with no visible skin irritation observed, and exhibited in vitro antioxidant activity. BGD inhibited cellular tyrosinase activity (4.86–35.82%) and melanin production (17.54–45.29%). These effects were accompanied by decreased expression of microphthalmia-associated transcription factor (MITF), tyrosinase (TYR), and dopachrome tautomerase (DCT) (*p* < 0.01). Furthermore, BGD increased the expression of nuclear factor erythroid 2-related factor 2 (Nrf2), heme oxygenase-1 (HO-1), and NAD(P)H:quinone oxidoreductase 1 (NQO1), while decreasing Kelch-like ECH-associated protein 1 (Keap1) expression (*p* < 0.01). It also lowered intracellular reactive oxygen species (ROS) levels and improved biochemical indicators associated with redox imbalance (*p* < 0.01), suggesting a potential role in alleviating H_2_O_2_-induced cellular oxidative stress through modulation of the Nrf2/Keap1 pathway.

## 1. Introduction

Skin serves as an important barrier against environmental insults. Long-term contact with external stressors, including ultraviolet irradiation and atmospheric contaminants, can disrupt skin homeostasis and induce a series of cellular and tissue injuries, thereby contributing to skin aging and hyperpigmentation [1]. These external insults may stimulate reactive oxygen species (ROS) formation within cutaneous cells. If ROS accumulation overwhelms the intrinsic antioxidant system, cellular redox homeostasis is disrupted, resulting in oxidative stress [2]. Persistent oxidative stress may damage cellular biomolecules and induce inflammatory responses and cellular dysfunction [3], ultimately accelerating skin aging.

Environmental insults may also disturb normal cutaneous pigment metabolism, leading to excessive melanin production and deposition. Melanin is an important biological pigment that absorbs ultraviolet (UV) radiation and provides a certain degree of photoprotection. Nevertheless, an overproduction or uneven deposition of melanin may contribute to skin hyperpigmentation [4]. Melanogenesis is regulated by numerous signaling molecules, transcription factors, and melanogenic enzymes. Microphthalmia-associated transcription factor (MITF) plays a pivotal role in controlling pigment production. It serves as an important transcriptional link between upstream signaling events and genes associated with melanin biosynthesis [5]. Tyrosinase (TYR) plays a central role in melanogenesis and is closely associated with the regulation of melanin production [6]. Dopachrome tautomerase (DCT) participates in the conversion of intermediate products during melanogenesis [7]. Persistent stimulation of these melanogenic processes may lead to excessive melanin production and subsequent hyperpigmentation. Therefore, modulation of melanogenesis represents an important strategy for managing abnormal skin pigmentation.

Black ginseng is a processed ginseng product traditionally prepared through repeated steaming and drying cycles and is rich in rare ginsenosides, polysaccharides, phenolic compounds, and other bioactive constituents [8]. Accumulating evidence has highlighted the biological potential of black ginseng. Its reported activities include antioxidant, anti-inflammatory, and anti-aging properties, which have led to growing interest in its potential applications in skin research [9,10]. Deer antler refers to the unossified or incompletely ossified antlers of male sika deer (Cervus nippon) or red deer (Cervus elaphus). Notably, deer antlers undergo annual regeneration, making them a naturally renewable animal-derived resource. Deer antler peptides are a group of bioactive peptides derived from deer antler and have demonstrated antioxidant and anti-aging properties, as well as the ability to promote cell proliferation and tissue repair, suggesting their potential applications in skin repair and skincare [11,12]. Different natural bioactive ingredients can exhibit enhanced biological activities when used in combination. For example, the combined use of red ginseng and deer antler extracts has been reported to provide stronger protection against ultraviolet B (UVB)-induced skin damage than either extract alone [13]. Given their distinct chemical compositions and complementary biological activities, combining black ginseng with deer antler peptides may provide a basis for their application in skin care. With the growing interest in natural functional ingredients for skincare products, increasing attention has also been paid to the development of appropriate formulations. Emulsions are among the most widely used formulations in cosmetics due to their favorable stability and application properties. They can provide a suitable dispersion system for the incorporation and delivery of bioactive ingredients [14,15], thereby supporting their stability and functional performance in the formulation.

However, whether black ginseng extract and deer antler peptides can be formulated into a stable composite emulsion and whether the resulting formulation exhibits antioxidant and antimelanogenic activities have not yet been systematically investigated. Therefore, in the present study, BGD was prepared using black ginseng extract and deer antler peptides as the primary functional ingredients. Unlike previous studies that have mainly focused on the functional properties of individual ingredients, this study extends the investigation from individual ingredients to a composite formulation and further links chemical antioxidant capacity with cellular protection against oxidative stress and regulation of melanogenesis. On this basis, the physicochemical properties, antioxidant activity, and antimelanogenic activity of BGD were systematically evaluated, and its potential underlying mechanisms were further explored. This study thus expands the potential combined use of black ginseng extract and deer antler peptides and provides a basis for the functional evaluation of multifunctional composite emulsions.

## 2. Materials and Methods

### 2.1. Materials and Reagents

Black ginseng was sourced from Yanbian Hangongfang Health Products Co., Ltd. (Yanji, China) and Deer antler peptides were obtained as an enzymatically hydrolyzed peptide powder from deer antler from Shanghai Huajian Biotechnology Co., Ltd. (Shanghai, China). Butylene glycol, olive husk oil, and other emulsion ingredients were provided by Shandong Fengyuan Biotechnology Group Co., Ltd. (Jinan, Shandong, China). A ROS detection kit was obtained from Beyotime Biotechnology (Shanghai, China). 2,2-diphenyl-1-picrylhydrazyl (DPPH) and 2,2′-azinobis(3-ethylbenzothiazoline-6-sulfonic acid) (ABTS) were sourced from Shanghai Yuanye Biotechnology Co., Ltd. (Shanghai, China). Antibodies against TYR, MITF, and DCT were sourced from Abways (Shanghai, China). Assay kits for superoxide dismutase (SOD), catalase (CAT), malondialdehyde (MDA), and glutathione (GSH) were obtained from Nanjing Jiancheng Bioengineering Institute (Nanjing, China). Primary antibodies targeting β-actin, HO-1, Keap1, NQO1, and Nrf2 were provided by Cell Signaling Technology, Inc. (Danvers, MA, USA). The B16F10 cell line was obtained from Shanghai Zhongqiao Xinzhou Biotechnology Co., Ltd. (Shanghai, China).

### 2.2. Black Ginseng–Deer Antler Composite

Black ginseng was extracted twice with 75% ethanol (1:10, *w*/*v*) by ultrasound-assisted extraction (60 °C, 60 kHz, 40 min), followed by n-butanol partitioning and freeze-drying (pre-frozen at −80 °C for 24 h, condenser temperature <−40 °C, vacuum <10 Pa for 24 h) to obtain the black ginseng extract [16]. Based on preliminary experiments, black ginseng extract and deer antler peptides, both in powder form, were screened at different mass ratios for DPPH· scavenging activity, and a ratio of 3:2 was selected for subsequent emulsion preparation.

### 2.3. Formulation Optimization of the BGD

A Box–Behnken response surface design was applied, with the addition levels of the black ginseng–deer antler composite (A), butylene glycol (B), and olive husk oil (C) as the independent variables and the sensory score of the emulsion as the response. Sensory evaluation was performed by 10 laboratory members aged 20–30 years. The assessors were blinded to the identity of the formulations and evaluated the samples in a laboratory setting. Five attributes, including appearance, odor, spreadability, absorbability, and skin feel, were evaluated using a 9-point scale, with higher scores indicating better sensory performance. Each assessor evaluated each formulation once, and the scores for the five attributes were summed to obtain the overall sensory score. The specific scoring criteria are provided in Table S2. The factor levels are presented in Table 1, while the response surface design and results are presented in Table 2.

### 2.4. Preparation Process of the BGD

The oil phase consisted of olive husk oil, hydrogenated polyisobutene, and cetyl ethylhexanoate, while the aqueous phase contained the black ginseng–deer antler composite, butylene glycol, and deionized water. The aqueous phase was mixed using an AD500S-H high-speed homogenizer (Wiggens, Wuppertal, Germany) equipped with a propeller-type stirring head at 5000 rpm and room temperature until complete dissolution. Both phases were then heated to 75 °C, with the oil phase maintained until fully liquefied. The oil phase was slowly incorporated into the aqueous phase under continuous homogenization at 5000 rpm for 5 min at 75 °C to form a uniform emulsion. The resulting emulsion was subsequently subjected to vacuum deaeration for 10 min, transferred into sterilized containers, and sealed for storage.

### 2.5. Determination of Physicochemical Properties and Safety of BGD

#### 2.5.1. pH Measurement

The pH was measured according to GB/T 13531.1–2008 [17] using a calibrated pH meter at 25 °C. Each sample was measured in triplicate.

#### 2.5.2. Texture Profile Analysis

Texture properties were measured using a TA.XT ExpressC texture analyzer (Xiamen Chaoji Instrument Equipment Co., Ltd., Xiamen, China) with an S254ST probe. The pre-test, test, and post-test speeds were 1.0, 1.0, and 2.0 mm/s, respectively, with a trigger force of 10 g and a compression distance of 10 mm. Firmness, consistency, cohesiveness, and work of cohesion were recorded. Each sample was measured in triplicate.

#### 2.5.3. Stability Test

Stability was evaluated according to GB/T 29665–2013 [18]. Thermal and cold stability were assessed at 40 °C and −20 °C for 24 h, respectively, while centrifugal stability was evaluated at 1580× *g* for 30 min. Phase separation, oil separation, and color changes were recorded.

#### 2.5.4. Patch Test

A human patch test was conducted according to the Safety and Technical Standards for Cosmetics (2015 edition) [19]. Thirty volunteers aged 18–30 years were enrolled. Before participation, the volunteers were informed of the purpose and procedures of the test, the method of sample application, and the observation period. Participation was voluntary, and the volunteers were informed of their right to withdraw at any time. Oral informed consent was obtained from all participants before participation. The emulsion (0.020 g) was applied to the inner forearm for 24 h, with the opposite forearm serving as the control. After patch removal, skin reactions were assessed visually at 0.5, 24, and 48 h, and any visible erythema, edema, itching, or other adverse reactions were recorded.

### 2.6. Antioxidant Activity Assays

BGD was diluted with distilled water to obtain 2×, 5×, 10×, and 20× dilutions, while the undiluted BGD was designated as 1×. The resulting samples were used for the subsequent antioxidant activity assays. For all antioxidant assays, corresponding sample blanks were included to correct for the intrinsic color and turbidity of BGD, and the blank absorbance was subtracted from that of the corresponding sample reaction before calculation.

#### 2.6.1. DPPH· Radical Scavenging Assay

For each dilution, BGD (20 μL) was mixed with 180 μL of DPPH solution (0.1 mmol/L in ethanol) in a 96-well plate. The reaction mixture was kept away from light for 30 min, after which absorbance was recorded at 517 nm (ELISA Microplate Reader, BK-EL10C, BIOBASE, Jinan, China). Vitamin C (Vc) at the corresponding dilution levels was measured under the same conditions. Each sample was analyzed in triplicate [20]. A sample blank containing the corresponding BGD dilution and absolute ethanol was used to correct for the intrinsic color and turbidity of BGD. DPPH· scavenging activity was calculated as follows:

$$\mathrm{DPPH}\cdot \mathrm{radical scavenging rate}\left(\%\right)=\left(1-\frac{A_{i}-A_{j}}{A_{0}}\right)\times 100\%$$

where (A_i_), (A_j_), and (A_0_) represent the absorbances of the sample, sample blank, and reagent control, respectively.

#### 2.6.2. ABTS^+^ Radical Scavenging Assay

The ABTS radical cation (ABTS^+^) was prepared by dissolving 0.18 g of ABTS in 50 mL of distilled water, with 0.03 g of potassium persulfate subsequently added. After thorough mixing, the solution was kept at room temperature in the dark for 12 h to allow radical formation. Before the measurement, the prepared solution was brought to the required absorbance (0.70 ± 0.02 at 405 nm) by adding distilled water. For the scavenging assay, 270 μL of ABTS^+^ solution was dispensed into each well, followed by 30 μL of BGD at the respective dilution level. After a 6 min period under light-protected conditions, measurements were performed at 460 nm. Vc at the corresponding dilution levels was measured under the same conditions. All measurements were conducted in triplicate [21]. ABTS^+^ radical-scavenging activity was calculated as follows:

$${\mathrm{ABTS}}^{+}\;\mathrm{radical scavenging rate}\left(\%\right)=\left(1-\frac{A_{i}-A_{j}}{A_{0}}\right)\times 100\%$$

where (A_i_), (A_j_), and (A_0_) represent the absorbances of the sample reaction, sample blank, and reagent control, respectively.

#### 2.6.3. ·OH Radical Scavenging Assay

To evaluate hydroxyl radical (·OH) scavenging activity, 2 mL of BGD at each dilution level was combined with equal volumes (2 mL each) of 6 mmol/L FeSO_4_, 6 mmol/L H_2_O_2_, and 6 mmol/L salicylic acid prepared in ethanol. The reaction mixtures were incubated for 30 min. Their absorbance values were then determined at 510 nm. Vc at the corresponding dilution levels was tested in parallel. Each experimental condition was assessed in triplicate [22]. ·OH radical-scavenging activity was calculated as follows:

$$\cdot \mathrm{OH radical scavenging rate}\left(\%\right)=\left(1-\frac{A_{i}-A_{j}}{A_{0}}\right)\times 100\%$$

where (A_i_), (A_j_), and (A_0_) represent the absorbances of the sample reaction, sample blank, and reagent control, respectively.

#### 2.6.4. Total Antioxidant Capacity

For the FRAP assay, each dilution of BGD (60 μL) was combined with 6 mL of freshly prepared FRAP working reagent. After 10 min at 37 °C, the absorbance was measured at 593 nm. Vc at the corresponding dilution levels was measured under the same conditions. Each sample was tested three times [23]. FRAP values were determined using the calibration curve, y = 0.2169x + 0.1509 (R^2^ = 0.9965), as follows:

$$\mathrm{FRAP}\;(\mathrm{mmol}\cdot L^{-1})=\frac{A-0.1509}{0.2169}$$

### 2.7. Cell Culture

B16F10 cells were maintained in Dulbecco’s modified Eagle’s medium (DMEM). The medium contained 10% fetal bovine serum (FBS) and 1% penicillin-streptomycin and was kept under humidified conditions (37 °C, 5% CO_2_). Cells were used for subsequent experiments once approximately 80–90% confluence had been achieved.

### 2.8. Cell Viability Assay

B16F10 cells were seeded into 96-well plates (5 × 10^3^ cells/well) and cultured for 24 h to allow cell attachment. Blank and untreated control groups were established. Following the respective treatments, the Cell Counting Kit-8 (CCK-8) assay was used for cell viability assessment. A 10 μL aliquot of the reagent was applied to each well. The plates were maintained for an additional 3 h, after which readings were obtained at 450 nm (BIOBASE ELISA Microplate Reader, BK-EL10C) [24].

### 2.9. Melanin Content Assay

B16F10 cells were seeded into 6-well plates at 5 × 10^5^ cells/well. Once the cells had adhered, α-MSH was omitted from the Control group, whereas the remaining groups received 1 μmol/L α-MSH together with either BGD (1, 3, or 6 mg/mL) or Arbutin (0.1 mg/mL). After 24 h, cells were collected and resuspended in 1 mol/L NaOH supplemented with 10% dimethyl sulfoxide (DMSO). The suspensions were placed in an 80 °C water bath for 1 h to facilitate cell lysis [25]. The lysates were then transferred to a 96-well plate. Absorbance was measured at 475 nm.

### 2.10. Tyrosinase Activity Assay

Following the treatment described in Section 2.9, the cells were harvested and lysed in 200 μL of radioimmunoprecipitation assay (RIPA) lysis buffer. After three freeze–thaw cycles (−80 °C, 30 min/cycle) to facilitate cell lysis and the release of intracellular tyrosinase, the lysates were centrifuged using a BOLXJ H5424R refrigerated centrifuge (Shandong Baiou Medical Technology Co., Ltd., Jinan, China) at 9750× *g* for 10 min at 4 °C. The supernatants were collected. A 150 μL volume of 1 mg/mL L-3,4-dihydroxyphenylalanine (L-DOPA) was mixed with 50 μL of the sample in a 96-well plate. The reaction was protected from direct light and maintained for 30 min at 37 °C, followed by absorbance measurement at 475 nm [26].

### 2.11. Intracellular ROS Detection

Once the cells had adhered, the Control group was not exposed to H_2_O_2_, whereas the remaining groups were treated with 1.5 mmol/L H_2_O_2_ for 4 h. BGD (1, 3, or 6 mg/mL) or Vitamin C (1 mg/mL) was then added to the corresponding groups. After 24 h, intracellular ROS levels in B16F10 cells were assessed with the fluorescent probe 2′,7′-dichlorodihydrofluorescein diacetate (DCFH-DA). The cells were exposed to 5 μM DCFH-DA at 37 °C for 20 min. Excess probe was subsequently eliminated by rinsing the cells with PBS. Fluorescence images were acquired using an inverted fluorescence microscope. ImageJ (version 1.54g) was subsequently used to quantify the fluorescence intensity [27].

### 2.12. Determination of Oxidative Stress-Related Parameters

Following the treatment described in Section 2.11, B16F10 cells underwent lysis in RIPA buffer on ice. The lysates were centrifuged using a BOLXJ H5424R refrigerated centrifuge at 9750× *g* for 10 min at 4 °C, and the supernatants were collected for subsequent biochemical measurements. Commercial assay kits were used to determine SOD, CAT, MDA, and GSH levels in accordance with the instructions provided by the manufacturers.

### 2.13. Western Blotting

Protein content was quantified using the bicinchoninic acid (BCA) assay. Equal protein loading was achieved before denaturation at 90 °C, followed by separation using sodium dodecyl sulfate–polyacrylamide gel electrophoresis (SDS-PAGE) with an 8% gel (Solarbio, Cat. No. P1200). The resulting protein bands were transferred onto membranes by wet electroblotting. The membranes underwent blocking with 5% non-fat milk for 1 h, followed by overnight exposure to the respective antibodies at 4 °C. After washing, secondary antibodies were added for 2 h at room temperature. Protein bands were visualized by chemiluminescence using a UVP ChemStudio Imaging System (Analytik Jena, Jena, Germany). Densitometric analysis was performed with ImageJ (version 1.54g) software [28].

### 2.14. Statistical Analysis

Data analysis and visualization were conducted using GraphPad Prism 8.0. One-way analysis of variance (ANOVA) followed by Dunnett’s multiple comparisons test was used for multi-group comparisons. Data are presented as mean ± standard deviation (SD) from three technical replicates (*n* = 3), and *p* < 0.05 was considered statistically significant.

## 3. Results

### 3.1. Results of BGD Formula Optimization

The ANOVA results for the Box–Behnken response surface model are summarized in Table 3. The fitted model showed a high F-value of 105.40 (*p* < 0.001). Conversely, the lack-of-fit term was insignificant (*p* > 0.05), confirming the adequacy of the fitted model. Based on the F-values, the effects of the three factors on the sensory score followed the order of olive husk oil (C) > butylene glycol (B) > black ginseng–deer antler composite (A), and all quadratic terms were highly significant (*p* < 0.01). The fitted quadratic model was described as follows:

Y = 35.60 − 1.71A − 3.00B − 8.44C − 1.48AB − 1.45AC + 0.725BC − 5.28A^2^ − 6.85B^2^ − 7.93C^2^ (1)

**Figure 1:**
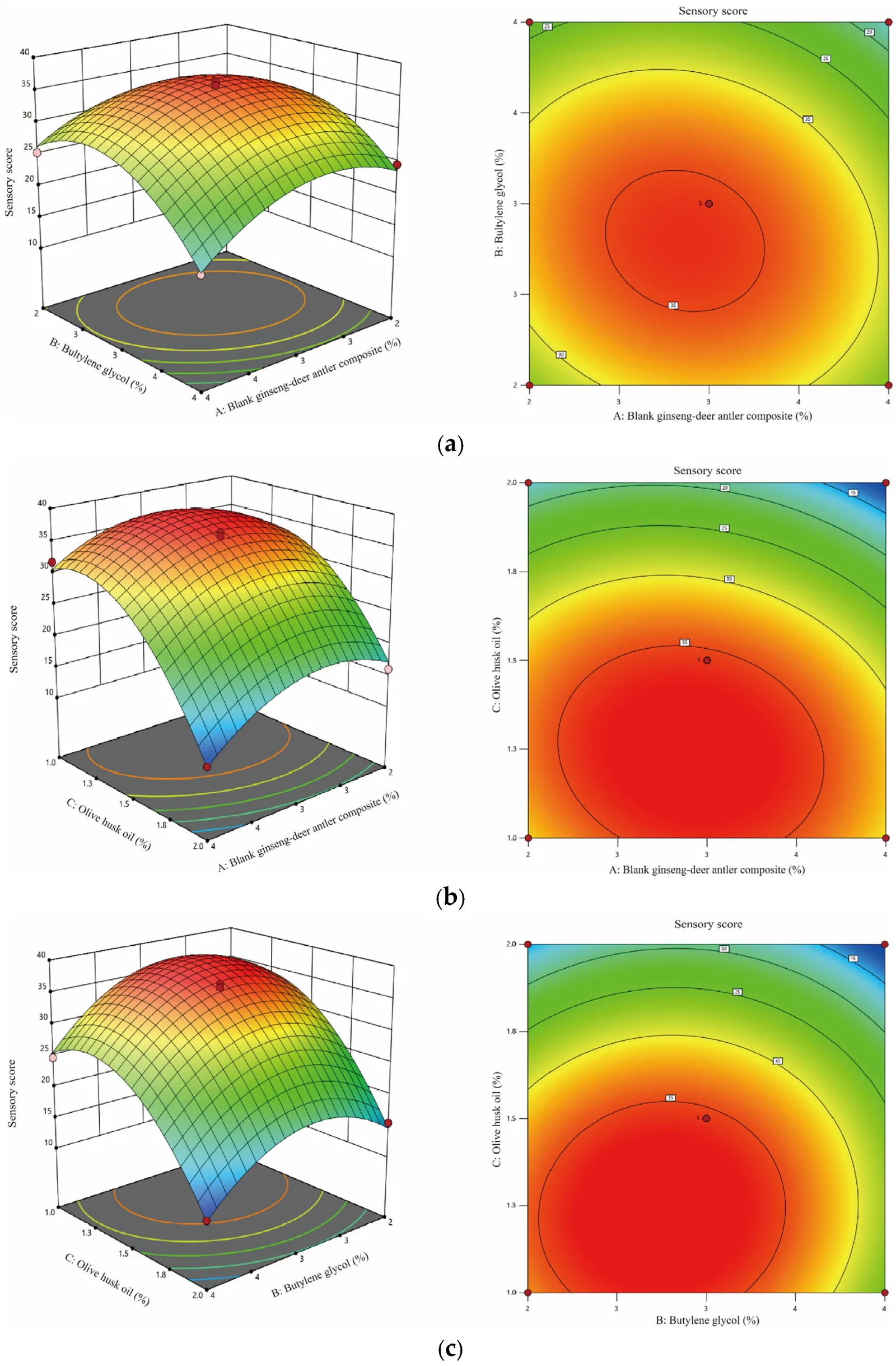
Response surface analysis of the optimized formulation. (**a**) Interaction between the black ginseng–deer antler composite and butylene glycol. (**b**) Interaction between the black ginseng–deer antler composite and olive husk oil. (**c**) Interaction between butylene glycol and olive husk oil.

Response surface analysis was subsequently performed to illustrate the effects of interactions among the independent variables (Figure 1). Significant interactions were observed between the black ginseng–deer antler composite and butylene glycol (A × B) and between the black ginseng–deer antler composite and olive husk oil (A × C) (*p* < 0.05), while the butylene glycol-olive husk oil interaction (B × C) showed a relatively limited effect. Based on the response surface optimization, the optimal formulation predicted by the model consisted of 3.0% black ginseng–deer antler composite, 2.0% butylene glycol, and 1.3% olive husk oil, with a predicted sensory score of 37.2. Validation of the optimized formulation yielded an actual sensory score of 38.5 ± 1.35. The absolute difference between the predicted and experimental values was 1.3 points, corresponding to a relative difference of 3.49%. These findings support the predictive performance of the established model.

### 3.2. Physicochemical Properties and Safety of BGD

#### 3.2.1. Texture and Physicochemical Properties of BGD

As shown in Table 4, BGD exhibited favorable textural properties, indicating an appropriate consistency, texture, and sensory feel. Its pH was close to the normal physiological pH range of human skin. No phase separation or emulsion breakage was observed after centrifugation or 24 h of thermal and cold treatments. BGD showed no apparent physical instability under the tested conditions.

#### 3.2.2. Safety Evaluation of BGD

The patch test results are presented in Table 5. None of the 30 participants developed skin irritation or other adverse reactions at 0.5, 24, or 48 h after patch application, indicating good skin compatibility of BGD under the test conditions.

### 3.3. Antioxidant Activity of BGD

**Figure 2:**
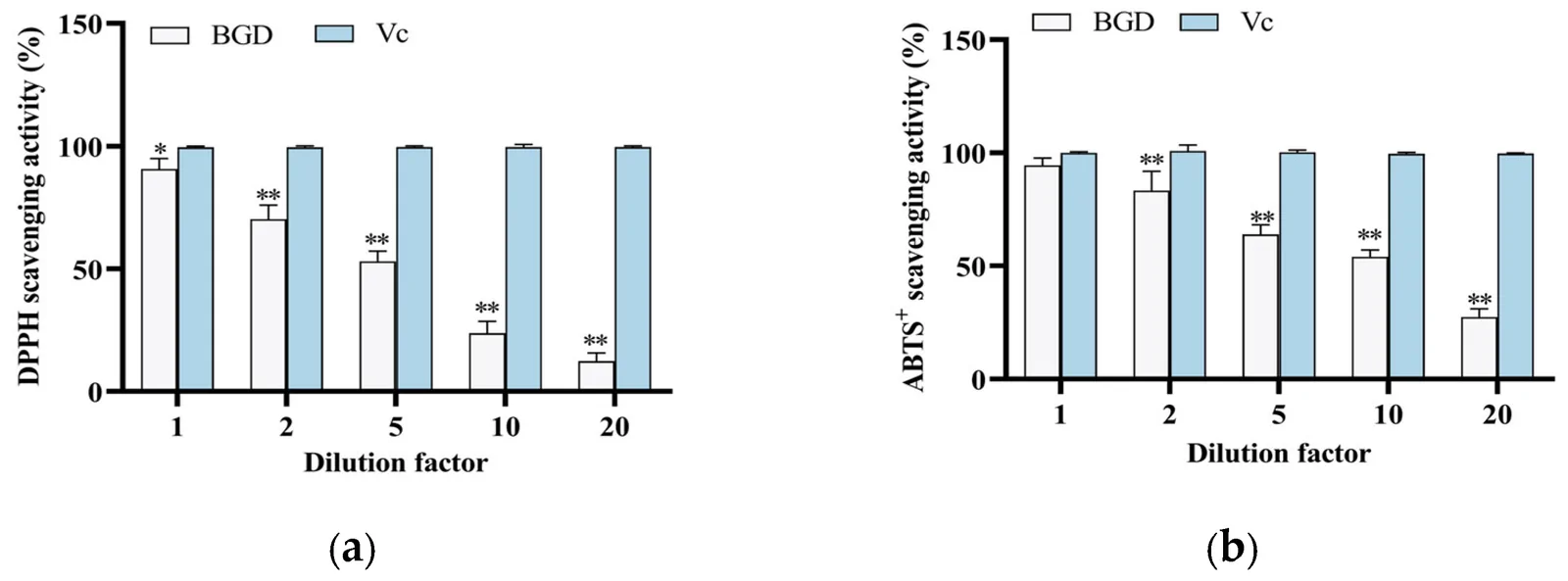

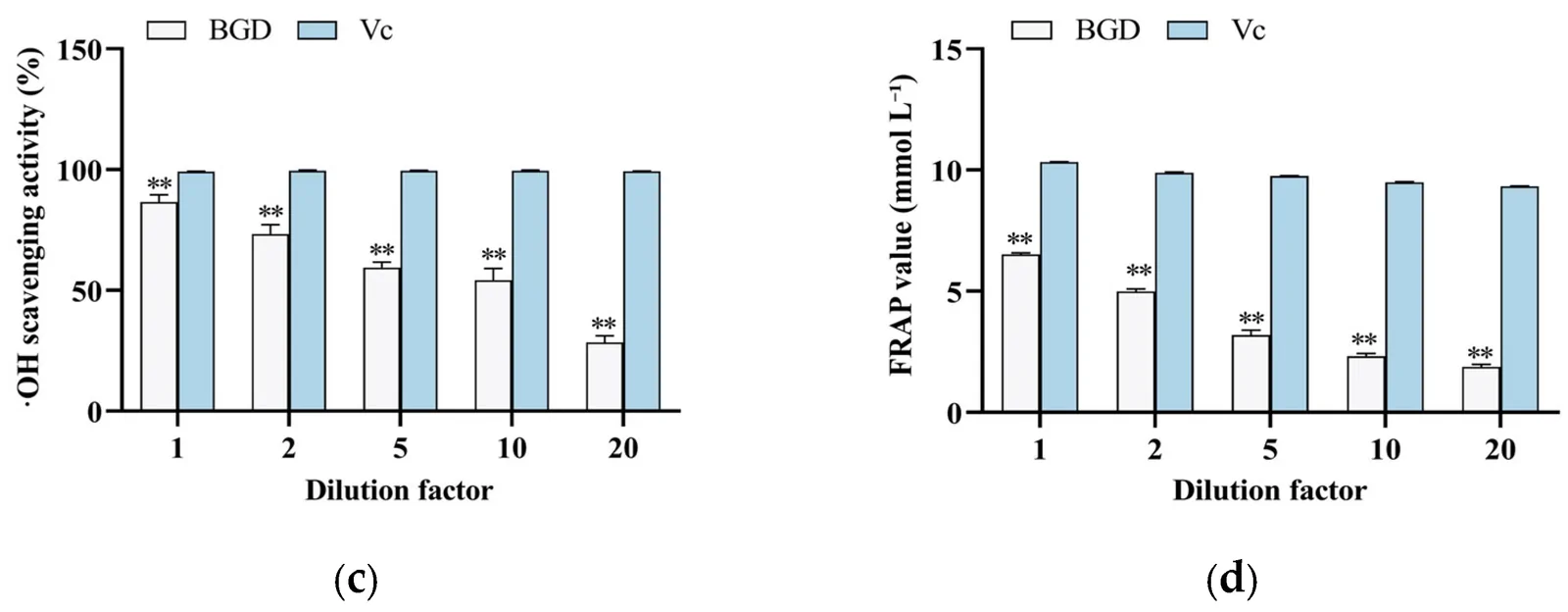
Antioxidant capacity of BGD in vitro. (**a**) DPPH· scavenging activity. (**b**) ABTS^+^ scavenging activity. (**c**) ·OH scavenging activity. (**d**) FRAP value. Results are presented as mean ± SD (*n* = 3). * *p* < 0.05 and ** *p* < 0.01, vs. the corresponding Vc group.

As shown in Figure 2, BGD exhibited scavenging activity against DPPH·, ABTS+, and ·OH across the tested dilution range. The scavenging activities increased with decreasing dilution, indicating a concentration-dependent response. The highest scavenging rates for DPPH, ABTS^+^, and ·OH were 90.60 ± 3.53%, 94.38 ± 2.73%, and 86.57 ± 2.47%, respectively. At a dilution factor of 1, BGD exhibited the highest FRAP, with a value of 6.514 ± 0.072 mmol/L. Overall, BGD showed strong free-radical scavenging activity, together with considerable reducing power, demonstrating its potent in vitro antioxidant activity.

### 3.4. Effects of BGD on Melanogenesis in B16F10 Cells

#### 3.4.1. Effects of BGD on Cell Viability and Melanogenesis in B16F10 Cells

**Figure 3:**
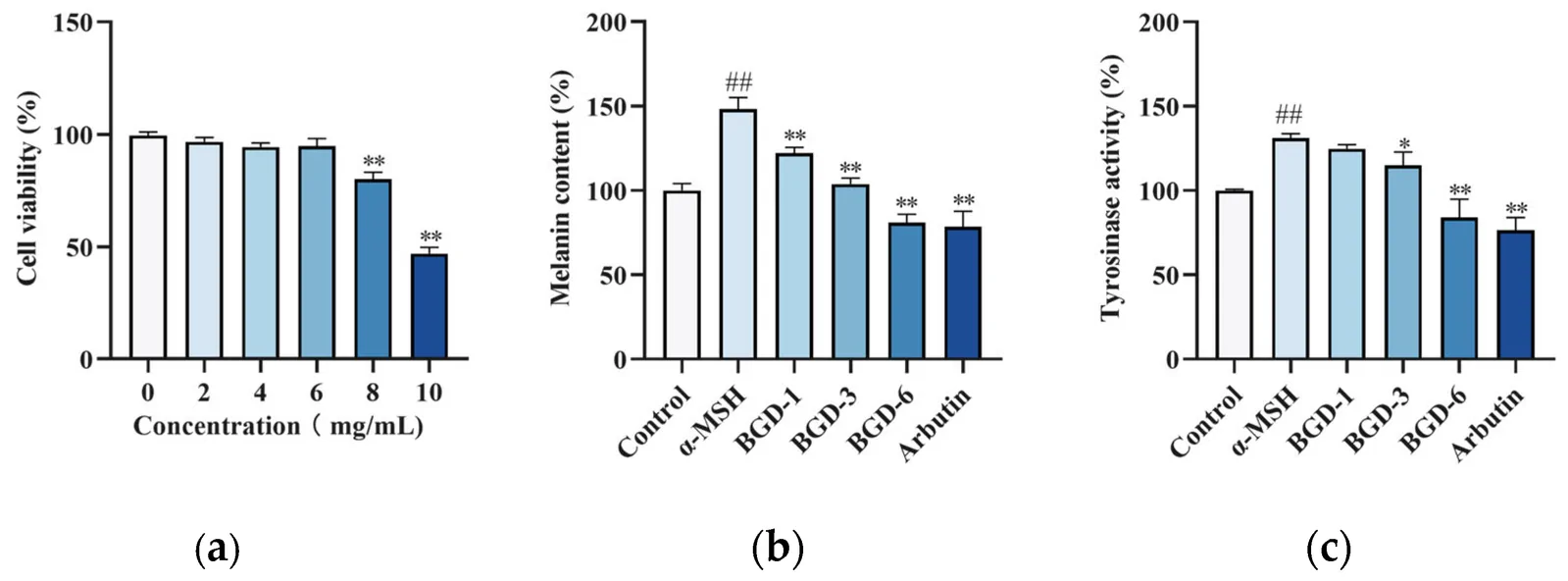
Effects of BGD on melanogenesis in B16F10 cells. (**a**) Different concentrations of BGD (0, 2, 4, 6, 8, 10 mg/mL). (**b**) Melanin content. (**c**) Tyrosinase activity. Results are presented as mean ± SD (*n* = 3). ^##^ *p* < 0.01 vs. Control; * *p* < 0.05 and ** *p* < 0.01, vs. α-MSH.

To determine a suitable concentration range, BGD was initially tested across a range of concentrations in B16F10 cells, and cell viability was subsequently assessed. As illustrated in Figure 3a, no obvious loss of viability was observed at concentrations up to 6 mg/mL, with viability values remaining above 90%. A marked loss of cell viability was observed following treatment with 8 mg/mL BGD (*p* < 0.01). Based on these findings, BGD was tested at 1, 3, and 6 mg/mL in subsequent experiments.

Figure 3b,c show that α-MSH exposure markedly increased cellular melanin content and tyrosinase activity relative to the Control group (*p* < 0.01), demonstrating successful establishment of the melanogenesis model. Treatment with BGD progressively attenuated both responses as the concentration increased. At 6 mg/mL, BGD produced inhibition rates of 45.29% for melanin formation and 35.82% for tyrosinase activity. Neither parameter varied significantly from that observed with Arbutin (*p* > 0.05). The concurrent decreases in melanin content and tyrosinase activity may be associated with the inhibitory effect of BGD on tyrosinase activity, thereby reducing melanin synthesis and accumulation.

#### 3.4.2. Effects of BGD on Melanogenesis-Related Proteins

**Figure 4:**
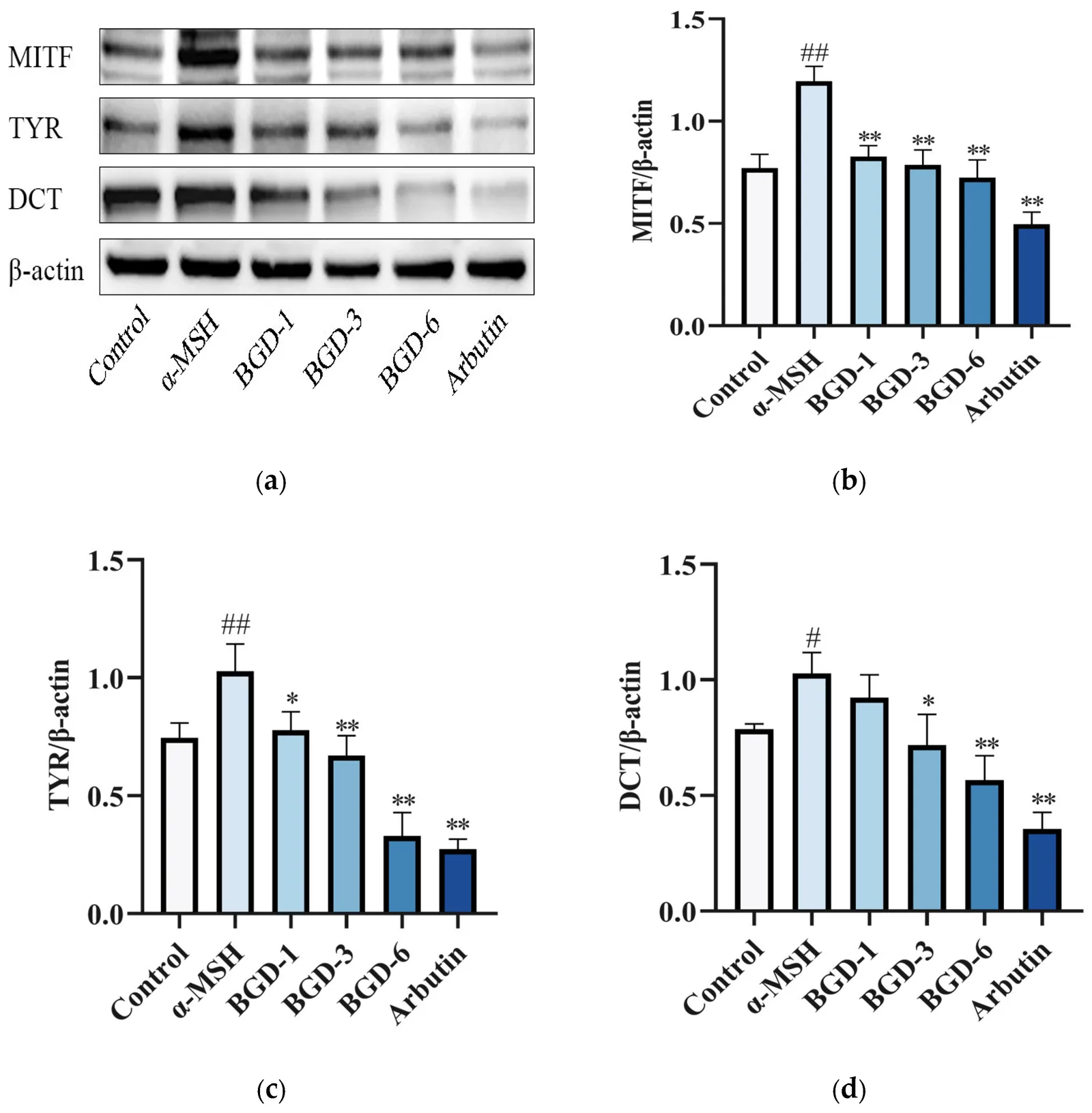
Effects of BGD on melanogenesis-related proteins in B16F10 cells. (**a**) Western blot bands. (**b**–**d**) Quantification of MITF, TYR, and DCT protein expression, respectively. Results are presented as mean ± SD (*n* = 3). ^#^ *p* < 0.05 and ^##^ *p* < 0.01 vs. Control; * *p* < 0.05 and ** *p* < 0.01 vs. α-MSH.

To further explore the molecular basis of the antimelanogenic effect of BGD, Western blotting was performed to evaluate melanogenesis-related protein expression in B16F10 cells. The results presented in Figure 4 demonstrate that α-MSH stimulation resulted in a significant elevation of MITF, TYR, and DCT protein levels, while the Control group exhibited relatively lower expression levels (*p* < 0.05). This increase may be attributed to α-MSH-induced stimulation of melanogenesis-related protein expression, thereby promoting melanin synthesis. BGD treatment suppressed the expression of these proteins to varying degrees, with their expression levels decreasing progressively with increasing BGD concentration. These findings suggest that the inhibitory effect of BGD on melanogenesis may be associated with reduced expression of MITF and its downstream melanogenesis-related proteins.

### 3.5. Effects of BGD on Oxidative Stress in B16F10 Cells

#### 3.5.1. Effect of H_2_O_2_ on B16F10 Cell Viability

**Figure 5:**
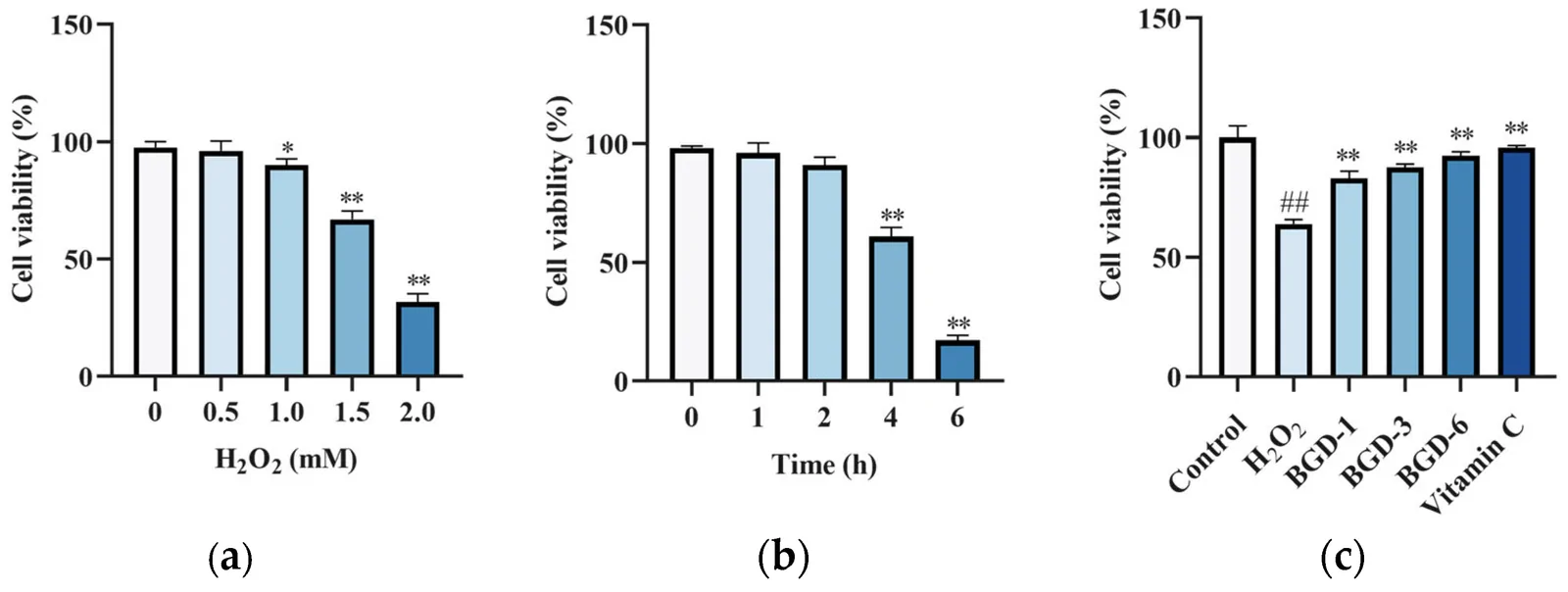
Effects of H_2_O_2_ exposure on B16F10 cell viability. (**a**) H_2_O_2_ concentrations of 0, 0.5, 1.0, 1.5, and 2.0 mM. (**b**) H_2_O_2_ exposure for 0, 1, 2, 4, and 6 h. * *p* < 0.05 and ** *p* < 0.01 indicate significant differences between groups. (**c**) Cell viability under different treatments. Results are presented as mean ± SD (*n* = 3). ^##^ *p* < 0.01 vs. Control; ** *p* < 0.01 vs. H_2_O_2_.

B16F10 cells received H_2_O_2_ stimulation at 0, 0.5, 1.0, 1.5, and 2.0 mM for 24 h. Subsequently, cells underwent treatment with the selected H_2_O_2_ concentration for 0, 1, 2, 4, or 6 h to determine suitable conditions for establishing oxidative stress. Results presented in Figure 5 reveal that increasing H_2_O_2_ concentrations led to a progressive decline in cell viability, indicating a dose-dependent cytotoxic effect of H_2_O_2_. Cell viability was significantly reduced after 4 h of H_2_O_2_ exposure (*p* < 0.01). Therefore, treatment with 1.5 mM H_2_O_2_ for 4 h was selected for oxidative stress model construction.

The cytoprotective effect of BGD following H_2_O_2_ exposure was then assessed. H_2_O_2_ stimulation caused a significant decline in B16F10 cell viability compared with untreated cells (*p* < 0.01), indicating the successful induction of cellular oxidative damage by H_2_O_2_. BGD treatment at the tested concentrations significantly improved cell viability (*p* < 0.01), suggesting that BGD alleviated H_2_O_2_-induced cellular injury.

#### 3.5.2. Effect of BGD on Intracellular ROS Levels

**Figure 6:**
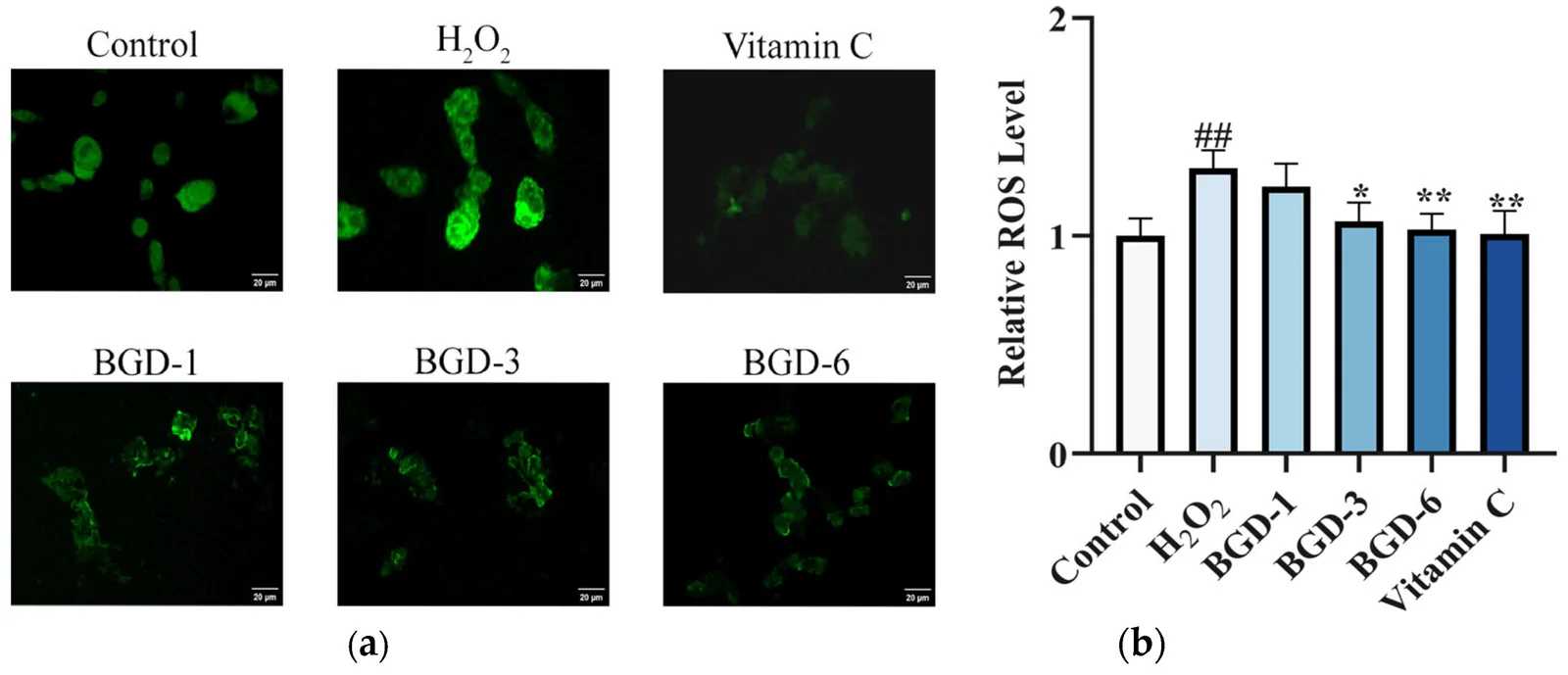
Effects of BGD on ROS levels in B16F10 cells (Scale bar = 20 um). (**a**) Representative fluorescence micrographs. (**b**) Fluorescence intensity quantification. Results are presented as mean ± SD (*n* = 3). ^##^ *p* < 0.01 vs. Control; * *p* < 0.05 and ** *p* < 0.01 vs. H_2_O_2_.

As illustrated in Figure 6, H_2_O_2_ exposure markedly enhanced intracellular green fluorescence intensity and elevated ROS levels compared with the Control group (*p* < 0.01), indicating substantial ROS accumulation. This increase may result from the elevated oxidative burden induced by exogenous H_2_O_2_, which disrupts the balance between ROS generation and clearance. BGD treatment markedly reduced the fluorescence intensity and intracellular ROS levels (*p* < 0.01), suggesting that BGD may alleviate H_2_O_2_-induced ROS accumulation.

#### 3.5.3. Effects of BGD on Oxidative Stress-Related Parameters

**Figure 7:**
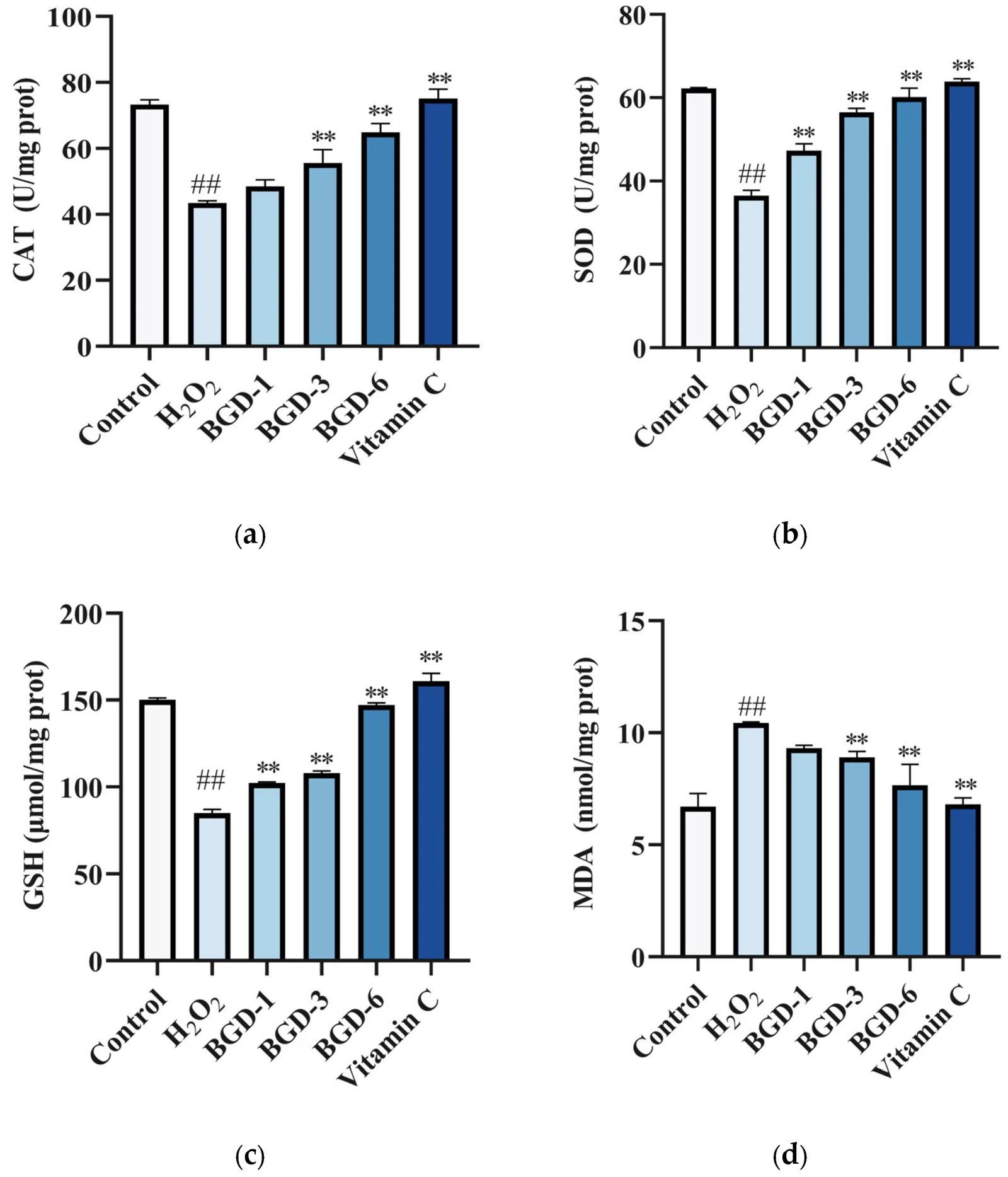
Effects of BGD on oxidative stress-related parameters in B16F10 cells. (**a**) CAT; (**b**) GSH; (**c**) SOD; (**d**) MDA. Results are presented as mean ± SD (*n* = 3). ^##^ *p* < 0.01 vs. Control; ** *p* < 0.01 vs. H_2_O_2_.

Figure 7 shows that high-dose BGD significantly elevated CAT and SOD activities as well as GSH levels (*p* < 0.01), whereas MDA levels were significantly decreased (*p* < 0.01) compared with the H_2_O_2_ group. These changes may be associated with enhanced endogenous antioxidant defenses and the clearance of oxidative products by BGD, thereby alleviating H_2_O_2_-induced lipid peroxidation and reducing MDA accumulation.

#### 3.5.4. Effects of BGD on Nrf2/Keap1 Signaling

**Figure 8:**
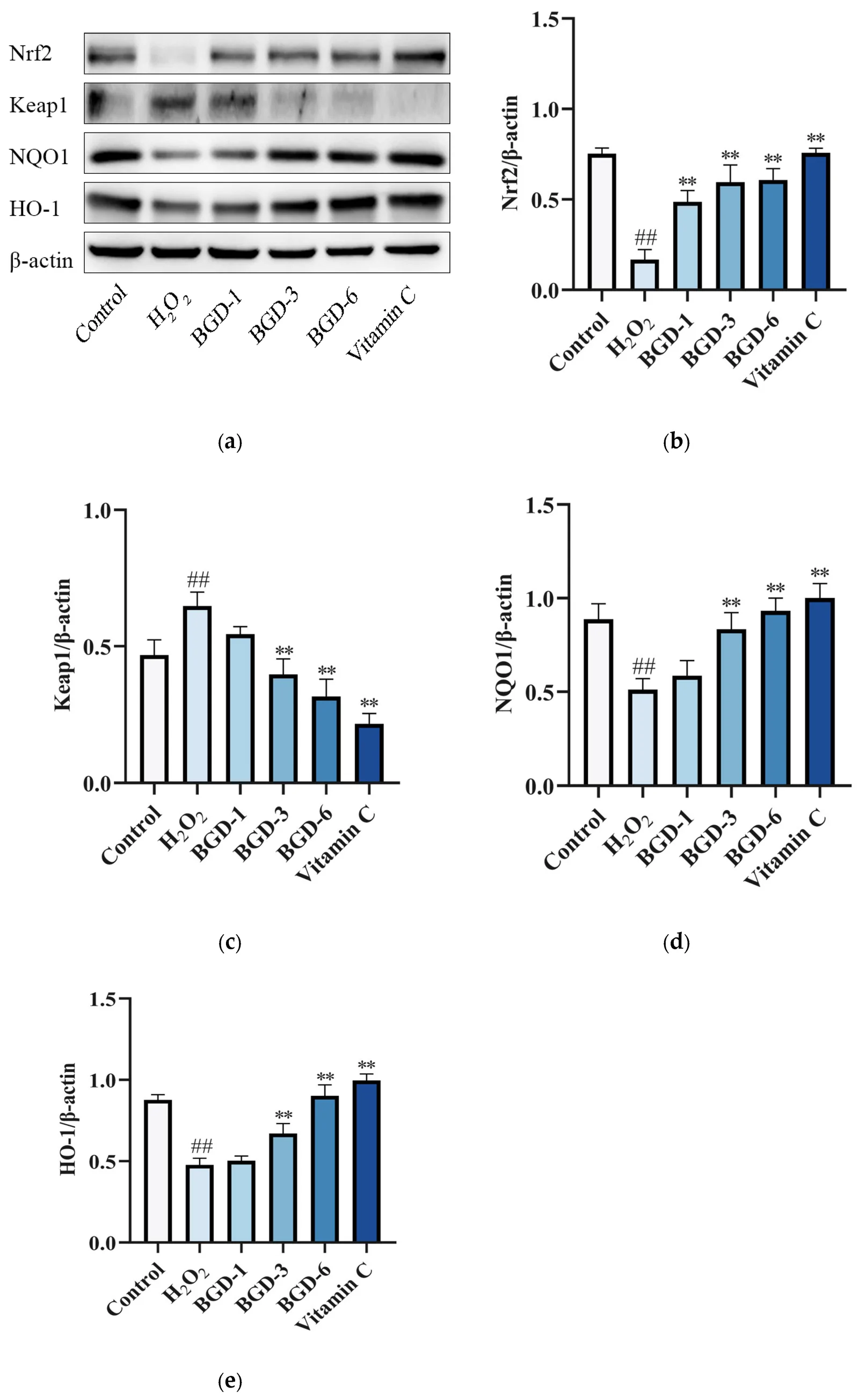
Effects of BGD on oxidative stress-related proteins in B16F10 cells. (**a**) Western blot bands. (**b**–**e**) Quantification of Nrf2, Keap1, NQO1 and HO-1 protein expression, respectively. Results are presented as mean ± SD (*n* = 3). ^##^ *p* < 0.01 vs. Control; ** *p* < 0.01 vs. H_2_O_2_.

To explore how BGD protects against oxidative stress, the expression levels of proteins involved in the Nrf2/Keap1 pathway were analyzed by Western blot analysis. Figure 8 shows that H_2_O_2_ exposure caused a marked reduction in Nrf2 protein expression relative to the Control group, accompanied by reduced expression of HO-1 and NQO1, as well as increased Keap1 expression (*p* < 0.01), suggesting that H_2_O_2_ may impair Nrf2-mediated antioxidant responses. Compared with H_2_O_2_-treated cells, BGD treatment dose-dependently decreased Keap1 expression while promoting Nrf2, HO-1, and NQO1 expression (*p* < 0.01). Overall, BGD partially reversed the H_2_O_2_-induced changes in Nrf2/Keap1 pathway-related protein expression, suggesting that its antioxidant effect may be associated with modulation of this signaling pathway.

## 4. Discussion

**Figure 9:**
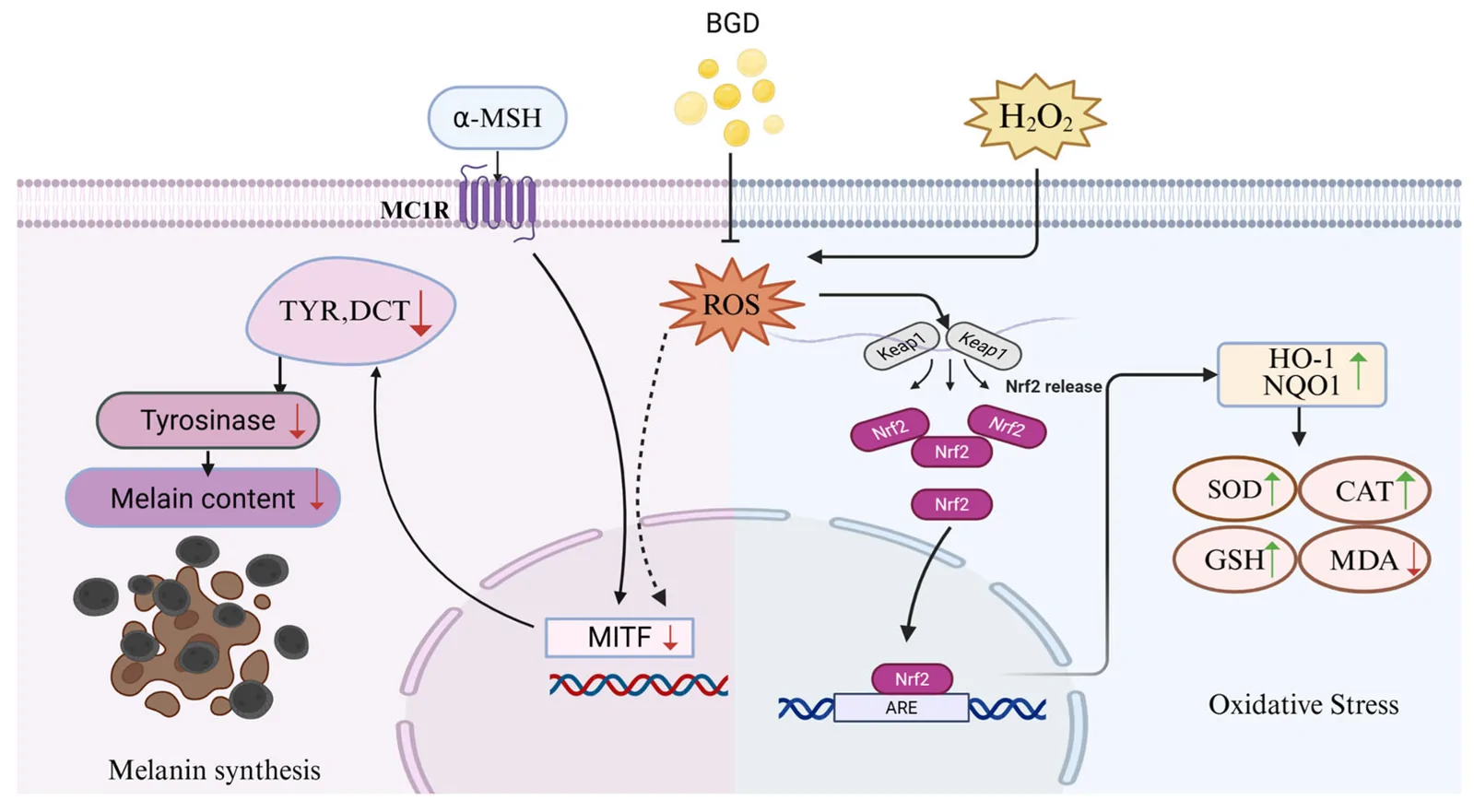
Schematic illustration of the antioxidant and antimelanogenic mechanisms of BGD.

The BGD prepared in this study exhibited favorable physicochemical properties and in vitro antioxidant activity. The composition and physicochemical properties of an emulsion can affect its stability and may also influence the dispersion and stability of bioactive components within the system [29]. In this study, response surface methodology was used to optimize the formulation, resulting in a relatively stable emulsion system, which may help maintain the stability of the bioactive components derived from black ginseng and deer antler peptides. Further in vitro antioxidant assays showed that BGD exhibited good scavenging activities against DPPH·, ABTS+, and ·OH, consistent with previous reports on the antioxidant activities of bioactive components from black ginseng and deer antler [30,31]. Both black ginseng and deer antler peptides contain various antioxidant-active components that may contribute to the antioxidant activity of BGD. Evaluating BGD at different dilution levels allowed its antioxidant activity to be characterized across a range of concentrations rather than at a single concentration. BGD retained considerable antioxidant activity across the tested dilution range, although the activity generally decreased with increasing dilution, indicating a concentration-dependent response. These findings provide a basis for further investigating the biological activities and underlying mechanisms of BGD at the cellular level. A schematic illustration of the antioxidant and antimelanogenic mechanisms of BGD is presented in Figure 9.

α-MSH is a commonly used inducer of melanogenesis. Upon binding to melanocortin 1 receptor (MC1R), α-MSH activates downstream signaling and promotes MITF expression, thereby upregulating the transcription of genes involved in melanogenesis [32]. In the present study, a B16F10 cell model with α-MSH-stimulated melanogenesis was established to evaluate the antimelanogenic activity of BGD. The inhibitory effects of BGD on melanin accumulation and tyrosinase activity became more pronounced as the treatment concentration increased. A similar effect has been reported for white mulberry fruit extract, which suppresses tyrosinase activity and melanin production in B16F10 cells [33]. Taken together, these findings indicate that inhibition of tyrosinase-related processes may contribute to the antimelanogenic effects of natural bioactive compounds.

Further analysis of melanogenesis-related proteins showed that BGD treatment markedly reduced the protein expression of MITF, TYR, and DCT. This finding suggests that the antimelanogenic effect of BGD may involve not only reduced tyrosinase activity but also the regulation of melanogenesis-related protein expression. Similar effects have been reported for cannabinol and *Siegesbeckia glabrescens* Makino flower absolute, both of which inhibit α-MSH-induced melanogenesis and reduce the expression of MITF and tyrosinase-related proteins, including TYR, TYRP1, and TYRP2 [34,35]. These findings suggest that MITF and its downstream tyrosinase-related proteins in the regulation of melanogenesis by natural bioactive compounds. Notably, both studies also reported alterations in mitogen-activated protein kinase (MAPK) signaling, although the specific pathways differed. Cannabinol mainly inhibited p38 mitogen-activated protein kinase (p38 MAPK) and c-Jun N-terminal kinase (JNK), whereas Siegesbeckia glabrescens Makino affected p38 MAPK, extracellular signal-regulated kinase 1/2 (ERK1/2), and JNK. Based on the present findings, the antimelanogenic effect of BGD may be associated with the regulation of MITF-mediated melanogenesis. However, the upstream molecular mechanisms involved remain to be elucidated.

To further evaluate its protective effect against oxidative stress, B16F10 cells were subjected to H_2_O_2_ exposure to induce oxidative stress. H_2_O_2_ can induce excessive intracellular accumulation of ROS, disrupt cellular redox homeostasis, and consequently cause cellular dysfunction [36]. ROS are important mediators of oxidative damage, and their excessive accumulation can further compromise endogenous antioxidant defenses and exacerbate cellular injury [37,38]. SOD, CAT, and GSH play important roles in maintaining cellular antioxidant capacity, whereas MDA is generated during membrane lipid peroxidation and is widely used to reflect oxidative damage to cell membranes [39]. In the present study, H_2_O_2_ treatment decreased cell viability and increased intracellular ROS levels, whereas BGD treatment restored cell viability and markedly reduced ROS accumulation. Further analysis showed that BGD enhanced the activities of SOD and CAT, while GSH was restored and MDA was reduced. These results suggest that the protective effect of BGD may involve reduced ROS accumulation, enhanced endogenous antioxidant defenses, and attenuated lipid peroxidation.

The Nrf2/Keap1 axis is a major regulator of cellular redox homeostasis and oxidative stress responses [40]. As a key antioxidant transcription factor, Nrf2 strengthens cellular antioxidant capacity through transcriptional control of a range of antioxidant genes [41]. Under oxidative stress, oxidative modification and conformational changes in Keap1 weaken its negative regulation of Nrf2, thereby promoting Nrf2 stabilization and nuclear translocation. Nrf2 subsequently activates antioxidant gene transcription through antioxidant response elements (AREs), leading to the expression of HO-1 and NQO1, thereby enhancing cellular resistance to oxidative insults [42,43]. In the present study, BGD treatment decreased Keap1 protein expression and increased the protein expression of Nrf2, HO-1, and NQO1. Consistent with our findings, a previous study reported that pretreatment with 6-Shogaol reversed the H_2_O_2_-induced decreases in Nrf2, HO-1, and NQO1 protein expression and alleviated oxidative damage in HEMn-MP cells, which was suggested to be associated with changes in the Nrf2-ARE signaling pathway [44]. Notably, that study mainly evaluated the Nrf2-ARE pathway based on changes in Nrf2 and its downstream proteins, HO-1 and NQO1, without further examining Keap1 expression. In contrast, the present study showed opposite changes in Keap1 and Nrf2, together with their downstream antioxidant proteins, providing additional evidence at the upstream regulatory level of the Nrf2 signaling axis. These findings further support the possibility that the antioxidant effects of BGD are associated, at least in part, with modulation of the Keap1/Nrf2 signaling pathway.

Notably, changes in cellular redox status may also be related to melanogenesis. Melanogenesis involves a series of redox reactions, and alterations in the intracellular redox environment may influence the signaling processes involved in melanogenesis. Therefore, antioxidant activity and the regulation of melanogenesis may not be entirely independent. A study of 3-O-ethyl ascorbic acid provides further evidence for this relationship. The study showed that 3-O-ethyl ascorbic acid attenuated ultraviolet A (UVA)-induced ROS accumulation, enhanced Nrf2-mediated antioxidant defenses, and inhibited α-MSH-induced melanogenesis. Moreover, silencing Nrf2 markedly attenuated its antimelanogenic effect, suggesting that redox regulation may contribute to the inhibition of melanogenesis [45]. Accordingly, the antioxidant and antimelanogenic effects of BGD may be related, with ROS and cellular redox status potentially representing a common regulatory link. BGD may alleviate oxidative damage by restoring cellular redox balance, while the improved redox status may also influence melanogenesis-related regulatory processes and consequently reduce melanin synthesis.

In addition, the antimelanogenic and antioxidant effects of BGD were evaluated using the murine B16F10 melanoma cell line, and further validation in human skin models is needed. The short-term stability tests provide limited information on long-term stability. Moreover, the individual contributions and potential synergistic effects of black ginseng extract and deer antler peptides in BGD remain to be clarified.

## 5. Conclusions

In conclusion, BGD was successfully prepared using an optimized formulation containing 3% black ginseng–deer antler peptide composite, 2% butylene glycol, and 1.3% olive husk oil, and exhibited good stability, with no visible skin irritation observed in the participant assessment. BGD demonstrated antioxidant activity through its DPPH·, ABTS^+^, and ·OH scavenging activities and ferric-reducing capacity. BGD may alleviate oxidative stress in cells, potentially through modulation of the Nrf2/Keap1 signaling pathway, thereby contributing to its antioxidant effects. In addition, BGD may exert antimelanogenic effects by modulating MITF-mediated melanogenesis. Collectively, BGD showed preliminary in vitro antioxidant and antimelanogenic effects and may have potential applications in cosmetic and functional skin-care products, providing experimental evidence for further investigation of black ginseng–deer antler peptide composite emulsions.

## Figures and Tables

**Table 1:** Levels of the independent variables.

Factors	Code	Actual Value of the Levels		
		−1	0	1
Black Ginseng–Deer Antler Composite (%)	A	2.0	3.0	4.0
Butylene Glycol (%)	B	2.0	3.0	4.0
Olive Husk Oil (%)	C	1	1.5	2.0

**Table 2:** Response surface design and results.

Run	A Black Ginseng–Peptide Deer Antler Composite (%)	B Butylene Glycol (%)	C Olive Husk Oil (%)	Sensory Score
1	3.0	3.0	1.5	34.30
2	2.0	2.0	1.5	27.00
3	4.0	2.0	1.5	25.30
4	3.0	4.0	1.0	24.70
5	3.0	2.0	1.0	32.80
6	4.0	3.0	1.0	31.70
7	2.0	4.0	1.5	24.60
8	3.0	2.0	2.0	15.50
9	3.0	3.0	1.5	35.00
10	2.0	3.0	2.0	16.00
11	3.0	3.0	1.5	36.20
12	3.0	4.0	2.0	10.30
13	4.0	4.0	1.5	17.00
14	3.0	3.0	1.5	35.70
15	4.0	3.0	2.0	10.90
16	3.0	3.0	1.5	36.80
17	2.0	3.0	1.0	31.00

**Table 3:** Regression model analysis of variance.

Source	Sum of Squares	Degrees of Freedom	Mean Square	F-Value	*p*-Value
Model	1329.01	9	147.67	105.40	<0.0001 **
A: Black Ginseng–Deer Antler Composite	23.46	1	23.46	16.75	0.0046 **
B: Butylene Glycol	72.00	1	72.00	51.39	0.0002 **
C: Olive Husk Oil	569.53	1	569.53	406.50	<0.0001 **
AB	8.70	1	8.70	6.21	0.0415 *
AC	8.41	1	8.41	6.00	0.0441 *
BC	2.10	1	2.10	1.50	0.2602 *
A^2^	117.16	1	117.16	83.62	<0.0001 **
B^2^	197.57	1	197.57	141.01	<0.0001 **
C^2^	264.44	1	264.44	188.74	<0.0001 **
Residual	9.81	7	1.40		
Lack of Fit	5.95	3	1.98	2.05	0.2490
Pure Error	3.86	4	0.97		
Cor Total	1338.82	16			
R^2^				0.9927	
R^2^adj				0.9833	

Note: * represents *p* < 0.05, ** represents *p* < 0.001.

**Table 4:** Texture characteristics and physicochemical properties of BGD.

Index	Result
Firmness (g)	24.83 ± 0.04
Consistency (g·sec)	146.93 ± 0.03
Cohesiveness (g)	9.28 ± 0.01
Work of Cohesion (g·sec)	17.38 ± 0.02
pH (25 °C)	6.65 ± 0.02
Thermal stability (40 °C, 24 h)	No phase separation
Cold stability (−20 °C, 24 h)	No phase separation
Centrifugal stability (1580× *g*, 30 min)	No phase separation

Note: Data are presented as mean ± SD (*n* = 3).

**Table 5:** Patch test.

Group	Number of Participants	Observation Time (h)	Number of Participants with Different Skin Reactions				
			−	±	+	++	+++
BGD group	30	0.5	30	0	0	0	0
		24	30	0	0	0	0
		48	30	0	0	0	0
Control group	30	0.5	30	0	0	0	0
		24	30	0	0	0	0
		48	30	0	0	0	0

## Data Availability

The original contributions presented in this study are included in the article and Supplementary Materials. Further inquiries can be directed to the corresponding authors.

## Supplementary Materials

The following supporting information can be downloaded at: https://www.mdpi.com/article/10.3390/foods15203665/s1, Table S1. Composition of the BGD Emulsion. Table S2. Criteria for sensory evaluation of BGD. Table S3. Information on antibodies used for Western blot analysis.

## Abbreviations

The following abbreviations are used in this manuscript:

BGD	Black ginseng–deer antler peptide composite emulsion
MITF	Microphthalmia-associated transcription factor
TYR	Tyrosinase
DCT	Dopachrome tautomerase
ROS	Reactive oxygen species
CAT	Catalase
SOD	Superoxide dismutase
GSH	Glutathione
MDA	Malondialdehyde
Nrf2	Nuclear factor erythroid 2-related factor 2
Keap1	Kelch-like ECH-associated protein 1
NQO1	NAD(P)H:quinone oxidoreductase 1
HO-1	Heme oxygenase-1
α-MSH	α-melanocyte-stimulating hormone
UV	Ultraviolet
UVB	Ultraviolet B
UVA	Ultraviolet A
DPPH	2,2-diphenyl-1-picrylhydrazyl
ABTS	2,2′-azinobis(3-ethylbenzothiazoline-6-sulfonic acid)
·OH	Hydroxyl radical
MC1R	Melanocortin 1 receptor
DMSO	Dimethyl sulfoxide
RIPA	Radioimmunoprecipitation assay
L-DOPA	L-3,4-dihydroxyphenylalanine
DCFH-DA	2′,7′-dichlorodihydrofluorescein diacetate
MAPK	Mitogen-activated protein kinase
JNK	c-Jun N-terminal kinase
ERK1/2	Extracellular signal-regulated kinase 1/2
