# Supplementary material for: Black Ginseng–Deer Antler Peptide Composite Emulsion: Antimelanogenic and Antioxidant Effects In Vitro
Source: Foods. 2026 Oct 15;15(20):3665. doi: 10.3390/foods15203665 (PMC13703655; doi:10.3390/foods15203665)
Supplement: Supplementary file 1 [file foods-15-03665-s001.zip › S3.pdf]

Table S3. Information on antibodies used for Western blot analysis.

Antibody	Host species	Supplier	Catalogue No.	Dilution
MITF	Rabbit	Abways	CY6661	1:1000
TYR	Rabbit	Abways	CY5555	1:1000
DCT	Rabbit	Abways	AY1921	1:800
Nrf2	Rabbit	Cell Signaling Technology	E5F1A	1:1000
Keap1	Rabbit	Cell Signaling Technology	P586	1:1000
HO-1	Rabbit	Cell Signaling Technology	E6Z5G	1:1000
NQO1	Rabbit	Cell Signaling Technology	D6H3A	1:1000
β-Actin	Rabbit	Abways	AB0035	1:10000
Anti-rabbit IgG, HRP-linked Antibody	Goat	Cell Signaling Technology	7074S	1:2000
