# Supplementary material for: Black Ginseng–Deer Antler Peptide Composite Emulsion: Antimelanogenic and Antioxidant Effects In Vitro
Source: Foods. 2026 Oct 15;15(20):3665. doi: 10.3390/foods15203665 (PMC13703655; doi:10.3390/foods15203665)
Supplement: Supplementary file 1 [file foods-15-03665-s001.zip › S1.pdf]

Table S1. Composition of the BGD Emulsion.

Component	Material number	Chemical name	Content (%)
A Aqueous phase	1	Acrylates	0.12
	2	Water	To 100
	3	1,2-Hexanediol	1.00
	4	Butylene Glycol	2.00
	5	Disodium EDTA	0.02
	6	Panthenol	0.30
	7	Black Ginseng-Deer Antler Peptide Composite	3.00
	8	Glycerol	4.00
	9	Xanthan gum	0.20
B Oil phase	1	Hydrogenated polyisobutene	3.00
	2	Cetyl ethylhexanoate	3.00
	3	Dimethicone	3.00
	4	Olive husk oil	1.30
	5	Glyceryl stearate	1.50
