# Supplementary material for: Black Ginseng–Deer Antler Peptide Composite Emulsion: Antimelanogenic and Antioxidant Effects In Vitro
Source: Foods. 2026 Oct 15;15(20):3665. doi: 10.3390/foods15203665 (PMC13703655; doi:10.3390/foods15203665)
Supplement: Supplementary file 1 [file foods-15-03665-s001.zip › S2.pdf]

Table S2. Criteria for sensory evaluation of BGD.

Evaluation Index	Evaluation Criteria	Score
Appearance	Uniform emulsion color and fine texture, with no visible phase separation, oil separation, or clumping, indicating good overall consistency.	1-9
Odor	Mild and natural odor, with no irritating or unpleasant odor; overall odor is harmonious and acceptable.	1-9
Spreadability	Easy to spread with uniform application and smooth spreading, without noticeable dragging or clumping.	1-9
Absorbability	Rapidly absorbed after application, with no obvious residue and a comfortable skin feel.	1-9
Skin feel	Leaves the skin moisturized and soft, without an oily or sticky sensation or a feeling of tightness, providing good overall comfort.	1-9
